# Glial cell line-derived neurotrophic factors (GFLs) and small molecules targeting RET receptor for the treatment of pain and Parkinson’s disease

**DOI:** 10.1007/s00441-020-03227-4

**Published:** 2020-06-17

**Authors:** Arun Kumar Mahato, Yulia A. Sidorova

**Affiliations:** Institute of Biotechnology, HiLIFE, University of Helsinki, Viikinkaari 5D, 00014 Helsinki, Finland

**Keywords:** Glial cell line-neurotrophic factor (GDNF), GDNF family ligands (GFLs), RET receptor, artemin (ARTN), Neurturin (NRTN), RET receptor tyrosine kinase, RET agonist, Small molecule, Drug design, Drug development

## Abstract

Rearranged during transfection (RET), in complex with glial cell line-derived (GDNF) family receptor alpha (GFRα), is the canonical signaling receptor for GDNF family ligands (GFLs) expressed in both central and peripheral parts of the nervous system and also in non-neuronal tissues. RET-dependent signaling elicited by GFLs has an important role in the development, maintenance and survival of dopamine and sensory neurons. Both Parkinson’s disease and neuropathic pain are devastating disorders without an available cure, and at the moment are only treated symptomatically. GFLs have been studied extensively in animal models of Parkinson’s disease and neuropathic pain with remarkable outcomes. However, clinical trials with recombinant or viral vector-encoded GFL proteins have produced inconclusive results. GFL proteins are not drug-like; they have poor pharmacokinetic properties and activate multiple receptors. Targeting RET and/or GFRα with small molecules may resolve the problems associated with using GFLs as drugs and can result in the development of therapeutics for disease-modifying treatments against Parkinson’s disease and neuropathic pain.

## Neurotrophic factors

Neurotrophic factors are a family of small secretory proteins which are necessary for survival and maintenance of both developing and mature neurons (Lanni et al. 2010). The key feature for the protein to be classified as a neurotrophic factor is the ability to promote the survival of certain neuronal population(s). Neurotrophic factors prevent neurodegeneration (Aron and Klein 2011), promote axonal growth (Kramer et al. 2006), and maintain synaptic plasticity and behavior (Lo 1995; Lewin and Barde 1996; Gómez-Palacio-Schjetnan and Escobar 2013). Neurotrophic factors are secreted into the extracellular space and, following neuronal innervation, they can be trafficked both in a retrograde and an anterograde manner (Altar and DiStefano 1998; Reynolds et al. 2000). Secreted neurotrophic factors act via receptors that are expressed in both peripheral and central nervous systems, activating intracellular signalling cascades important for neuronal survival and functioning (Chang et al. 2019).

Neurotrophic factors include neurotrophins, glial cell line-derived neurotrophic factor (GDNF) family ligands (GFLs), neurokines, and the mesencephalic astrocyte-derived neurotrophic factor/cerebral dopamine neurotrophic factor (MANF/CDNF) protein family. This review is focused on GFLs which are distant members of the transforming growth factor-β (TGF-beta) superfamily and their receptors. The GFLs consist of four proteins: GDNF, neurturin (NRTN), artemin (ARTN), and persephin (PSPN). They have conserved patterns of seven cysteine residues and function as homodimers (Eigenbrot and Gerber 1997). They all mainly transmit signals via transmembrane rearranged during transfection (RET) through binding to one of its preferential cognate co-receptors, GDNF family receptor alpha (GFRα), but can also act via neural cell adhesion molecule (NCAM) (Paratcha et al. 2003; Ilieva et al. 2019) and syndecan-3 (Bespalov et al. 2011).

## GDNF family ligand receptors

RET was identified as an oncogenic protein activated by chromosomal rearrangement (Takahashi et al. 1985; Takahashi and Cooper 1987; Takahashi 2001). RET has a unique extracellular domain consisting of four cadherin-like repeats, called cadherin-like domains CLD1–4, and a calcium-binding site between CLD2 and CLD3 (Anders et al. 2001). Calcium binding is necessary for the proper folding of RET and ligand binding (Anders et al. 2001; Ibáñez 2013). Proximal to the cadherin-like domains is the cysteine-rich region, which is further connected to the transmembrane domain. The cysteine-rich domain is necessary for ligand binding and proper protein confirmation (Amoresano et al. 2005). The transmembrane domain is required for dimerization of RET and is further linked to the intracellular domain. The intracellular domain consists of a juxtamembrane portion, a tyrosine kinase domain, and a C-terminal tail. Upon RET activation, multiple tyrosines in the RET intracellular domain become phosphorylated and serve as docking sites for adaptor proteins or enzymes needed for activation of downstream signalling cascades promoting cell growth, proliferation, survival, and differentiation (Airaksinen and Saarma 2002).

**Fig. 1:**
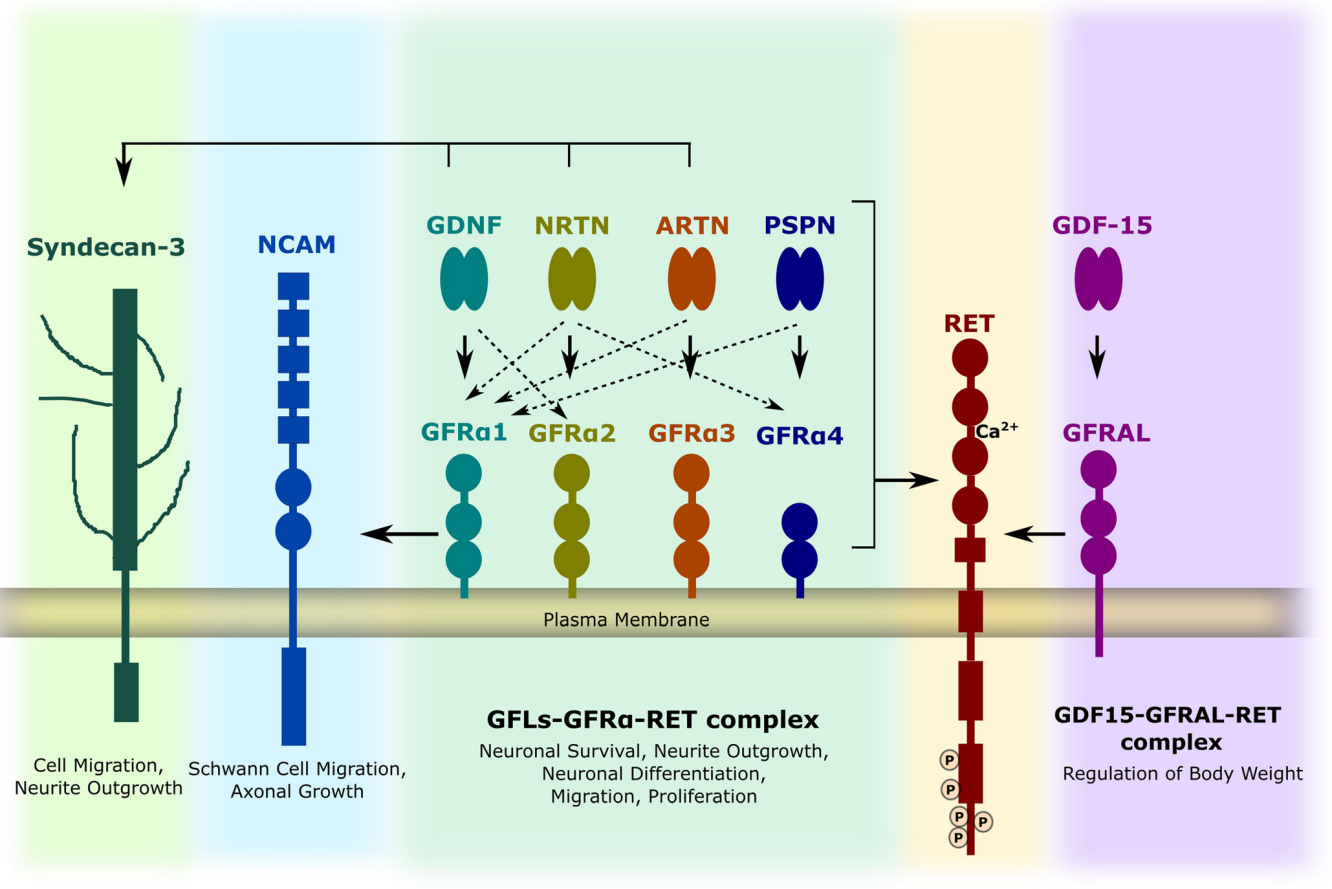
Neurotrophic factors and their receptors. GDNF family ligands and distant members of GFL include GDNF, NRTN, ARTN, PSPN, and GDF15. They all form homodimer and bind to respective co-receptor GFRα (1–4) and GFRΑL respectively. The complex of ligand and co-receptor interacts with RET which leads to phosphorylation of RET and activation of intracellular signalling cascades. GDNF can also signal through alternate receptor NCAM and syndecan-3 (GDNF, NRTN, and ARTN). GDNF signalling is important for survival, neurite outgrowth, migration, and differentiation of neuronal cells and regulation of body weight via appetite control

GFLs signal through transmembrane receptor tyrosine kinase RET via forming a tripartite complex. The signalling complex comprises two molecules of the RET receptor, two molecules of glycosylphosphatidylinositol-linked GFRα, and a dimeric ligand (Trupp et al. 1996; Durbec et al. 1996; Treanor et al. 1996). The presence of co-receptors provides specificity for ligand binding to a receptor complex. GDNF preferentially binds to GFRα1, NRTN to GFRα2, ARTN to GFRα3, and PSPN to GFRα4 (Airaksinen and Saarma 2002) (Fig. 1). However, there is cross-talk between ligand and co-receptors; for example, GDNF can also bind to GFRα2 and NRTN can in turn bind to GFRα1 (Bespalov and Saarma 2007). Further, there is experimental evidence that ARTN and PSPN can also interact with GFRα1 (Baloh et al. 1998; Sidorova et al. 2010).

GFLs may also signal RET-independently through NCAM which requires the presence of a GFRα co-receptor (Paratcha et al. 2003) and heparan sulfate proteoglycan syndecan-3 (Bespalov et al. 2011). The interaction of GFRα1 with NCAM downregulates NCAM-mediated cell adhesion independently of GDNF (Ibáñez 2010). The presence of GFRα1 potentiates the interaction of GDNF to NCAM and activates cytoplasmic tyrosine kinases Fyn and FAK which is necessary for Schwann cell migration and axonal growth in hippocampal and cortical neurons (Paratcha et al. 2003). Further, both NRTN and ARTN mediate release of immunoreactive calcitonin gene-related peptide (CGRP) through NCAM (Schmutzler et al. 2011). Syndecan-3 was found to be a receptor for GDNF, NRTN, and ARTN and promotes cell spreading and neurite outgrowth of hippocampal neurons independently from GFRα co-receptors (Bespalov et al. 2011).

Growth differentiation factor-15 (GDF-15), which is a distant member of the TGF-beta family, activates RET via forming a complex with GDNF receptor alpha-like (GFRΑL) and helps to regulate food intake, energy expenditure, and body weight (Hsu et al. 2017; Mullican et al. 2017; Yang et al. 2017), but may also play a role in the development and survival of dopamine and sensory neurons (Strelau et al. 2009).

## Parkinson’s disease

**Fig. 2:**
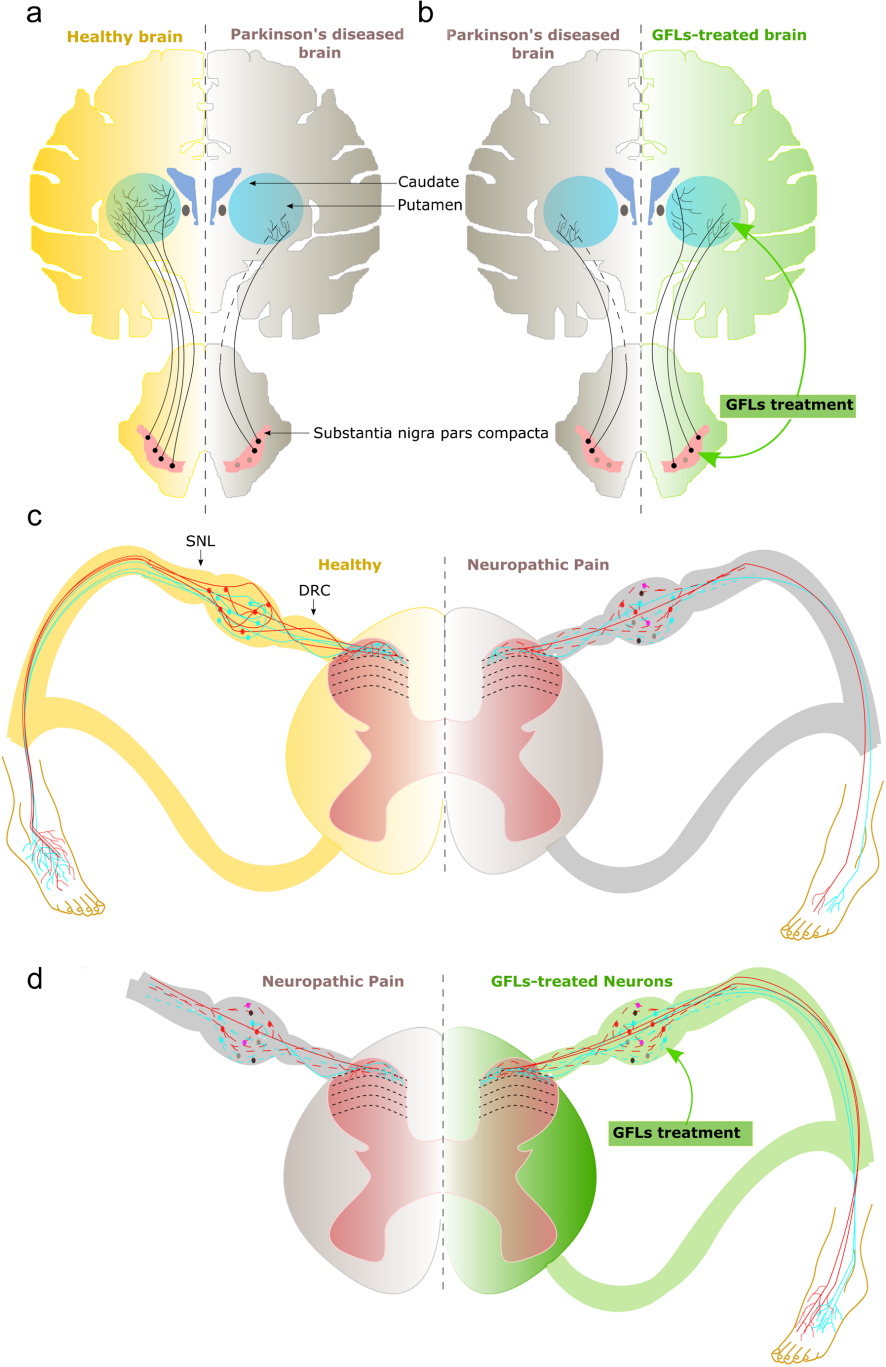
Schematic representation of healthy and degenerated neurons in both Parkinson’s disease and neuropathic pain. **a** Dopamine neuron projections in a healthy brain are shown in the left hemisphere (yellow) and in a brain of patient with Parkinson’s disease in the right hemisphere (gray). **b** GFL treatments simulate regrowth and restore dopamine fibers which are shown in the right hemisphere (green). **c** Sensory neurons locate in dorsal root ganglia and send their projections to periphery and to spinal cord in healthy people as shown on the right panel (yellow). **d** In neuropathic pain patients, sensory neurons degenerate and loose terminals in both periphery and in spinal cord as shown on the right panel (gray). Treatment with GFLs protects and restores central and peripheral projections of sensory neurons and targets them to topographically correct place in the spinal cord as shown on left panel (green). Arrow heads indicate the sites of lesions in spinal nerve ligation (SNL) model of NP and dorsal root crush (DRC) model of experimental neuropathy

Parkinson’s disease (PD) is the second most common neurodegenerative disease affecting 2–3% of the population ≥ 65 years of age (Poewe et al. 2017). The incidence of PD increases with age and the disease is more common in males (Miller and Cronin-Golomb 2010). It is a progressive neurodegenerative disorder which is characterized by the loss of dopamine neurons in the substantia nigra pars compacta (SNpc) (Dauer and Przedborski 2003). The loss of dopamine neurons leads to striatal dopamine deficiency causing motor impairment. The motor symptoms include bradykinesia, rigidity, resting tremor, and postural instability (Kravitz et al. 2010). PD is also associated with many non-motor symptoms such as hyposmia, depression, constipation, sleep disorders, pain, lack of motivation, and cognitive problems which increases disability for the patient (Postuma et al. 2012; Duncan et al. 2014). It is estimated that 30% of cell bodies and 80% of striatal dopamine axons are lost before the first symptoms of PD appear (Burke and O’Malley 2013) (Fig. 2). The molecular mechanism in the loss of dopamine neurons involves multiple pathways, which include α-synuclein (αSyn) proteostasis, mitochondrial function, oxidative stress, calcium homeostasis, axonal transport, and neuroinflammation (Poewe et al. 2017). The pathological hallmark feature of PD is the presence of Lewy bodies (LBs) in the brain. LB includes a variety of intracellular proteins, among which αSyn is the most abundant component of the inclusion (Spillantini et al. 1997). Recently, it was shown that LBs consist of a crowded mix of fragmented membranes, organelles, and vesicles and the process of LB formation is considered as the major process in the pathogenesis of PD (Shahmoradian et al. 2019). Currently, treatment of PD is based on pharmacological substitution of dopamine, administration of dopamine receptor agonists, and reduction of dopamine breakdown by supplying monoamine oxidase B inhibitors (Schapira and Olanow 2008). Further, deep brain stimulation is also recommended as an effective therapy for many neurological diseases including PD (Fang and Tolleson 2017). The available therapies can neither halt nor reverse the disease progression, have significant side effects, almost no effect on non-motor symptoms, and lose their efficacy with time. Therefore, there is an immense need to develop therapies for the disease-modifying management of PD.

## GFLs in Parkinson’s disease

GFLs are potent neurotrophic factors for the survival of dopamine neurons (Airaksinen and Saarma 2002). GDNF was the first member from the GFLs discovered to be the survival factor of dopamine neurons (Lin et al. 1993). NRTN, ARTN, PSPN, and GDF15 are also known to promote the survival of dopamine neurons both in vitro and in vivo (Horger et al. 1998; Strelau et al. 2000; Zihlmann et al. 2005). Comparative studies on various neurotrophic factors suggest that GDNF and NRTN are the most potent survival factors for dopamine neurons (Zihlmann et al. 2005). Moreover, the pattern of GFRα co-receptor expression and cross-reactivity of GFLs to GFRα co-receptors are in line with their survival-promoting properties in dopamine neurons. Therefore, both GDNF and NRTN have been extensively studied in various toxin-based models of PD in rodents and primates and showed remarkable neuroprotective and neurorestorative properties (Hoffer et al. 1994; Bowenkamp et al. 1995; Beck et al. 1995; Gash et al. 1996; Horger et al. 1998; Kirik et al. 2000; Kordower et al. 2000; Oiwa et al. 2002; Runeberg-Roos et al. 2016). Further, overexpression of both ARTN and PSPN in the mouse striatum and substantia nigra either by lentiviral gene transfer or by neural stem cell delivery prevents the degeneration of dopamine neurons (Rosenblad et al. 2000; Åkerud et al. 2002; Yin et al. 2014).

Human dopamine neurons highly express RET, GFRα1, and GFRα2 that are cognate receptors for GDNF and NRTN, respectively (Runeberg-Roos et al. 2016). In rodents, the expression of GFRα2 is much weaker compared to GFRα1 in the substantia nigra pars compacta (Horger et al. 1998; Wang et al. 2000). GFRα3 is expressed in peripheral nerves and ganglia, but not in the brain (Widenfalk et al. 1998; Naveilhan et al. 1998; Baloh et al. 1998). Functional GFRα4 is predominantly expressed in the thyroid gland (Lindahl et al. 2001; Vanhorne et al. 2005). These findings suggest that neuroprotective and neurorestorative effects of GFLs in the dopamine system are mostly mediated via GFRα1/RET and GFRα2/RET complexes.

Due to the remarkable support to dopamine neurons provided by GDNF and NRTN seen in both in vitro and in vivo studies, several clinical trials in PD patients were conducted. GFLs do not cross through the blood-brain barrier. Therefore, these proteins were delivered either as a purified form or by means of gene therapy directly into the brain using complex stereotactic surgery. In the first randomized double-blinded clinical trial, intracerebroventricularly delivered GDNF failed to produce clinical benefits. However, it was shown that in this study, GDNF was not able to reach the target tissues, thereby halting its therapeutic effects (Nutt et al. 2003). The next small-scale phase I/II clinical trials showed that the direct infusion of GDNF into the putamen was well-tolerated and improved motor function and increased [^18^F]DOPA uptake in the brains of PD patients (Gill et al. 2003; Slevin et al. 2005). However, the follow-up double-blinded randomized placebo-controlled trial with intraputaminal administration of GDNF failed to reach its primary efficacy endpoints (improvement in motor function) despite enhanced [^18^F]DOPA uptake in the putamen (Lang et al. 2006). The failure of this double-blinded trial can be explained by the poor tissue distribution of GDNF which might be due to the difference in the catheter size and pump design used for the study, and an insufficient number of patients in the treatment groups (Lang et al. 2006; Hutchinson et al. 2007). Furthermore, the study conducted in rhesus monkeys with the same delivery system as the one used in the double-blinded trial conducted by Lang and co-authors found that GDNF was unevenly distributed in the putamen, being highly concentrated around the catheter tip (Salvatore et al. 2006). The recent randomized placebo-controlled phase II study and extended treatment paradigm with intermittent intraputaminal convection-enhanced delivery of a low dose of GDNF also failed to reach the primary endpoint. Nevertheless, in this study, post hoc analysis revealed that 43% of the patients in the GDNF group showed a clinically significant motor improvement. Importantly, all GDNF-treated patients had increased [^18^F]DOPA uptake in the putamen. The results of these studies also suggested that the use of a higher dose of GDNF with 80 weeks of total study duration might result in a significant clinical benefit of GDNF (Whone et al. 2019b, a).

In order to achieve targeted therapy and continuous expression of GDNF throughout the target area, a viral vector-mediated delivery approach has been introduced. In a phase I open-label study, the adeno-associated virus (AAV) serotype 2-NRTN (CERE-120) delivery to putamen was safe and well-tolerated, and it improved motor function of PD patients (Marks et al. 2008). In a double-blinded sham surgery-controlled trial, injection of AAV2-NRTN bilaterally to putamina did not lead to a significant difference in primary endpoints in patients treated with CERE-120 compared to placebo-treated patients (Marks et al. 2010a, b). However, modest but significant benefits in the primary outcome compared with placebo controls were seen at 18 months for AAV2-NRTN treatment (Marks et al. 2010). Later on, immunohistochemical analysis of brains of PD patients and of non-human primates revealed the interspecies difference in the distribution pattern of NRTN in the SNpc. Even though striatal distribution between both species was comparable, the level of NRTN in the SNpc was drastically lower in humans compared to monkeys (Bartus et al. 2011). This can be explained by the impaired axonal transport which resulted in poor transfer of NRTN into the SNpc in PD patients. To test this hypothesis, an 18-month double-blinded, placebo-controlled trial in 51 patients with advanced PD with bilateral AAV2-NRTN injection into both the substantia nigra and putamen was conducted. However, no significant differences between groups in the primary endpoint or in most of the secondary endpoints were observed. The procedure was well-tolerated with no signs of serious adverse effects (Olanow et al. 2015). Nevertheless, post hoc analysis revealed a greater benefit in early-stage patients compared to advanced patients (Bartus and Johnson 2017). Furthermore, the treatment with AAV2-GDNF in a phase I clinical trial was found to be safe and well-tolerated, with increased putaminal distribution of GDNF and enhanced putaminal [^18^F]DOPA uptake suggesting neurotrophic effects on dopamine neurons (Heiss et al. 2019).

In summary, preclinical studies have demonstrated robust effects of GDNF family neurotrophic factors in a moderate neurotoxin animal model of PD. However, clinical trials with GDNF and NRTN have failed to demonstrate statistically significant benefits in PD patients. There might be several reasons behind the failure in translation of preclinical outcome. The tissue distribution of GFLs is limited because of its high binding affinity to heparan sulfate proteoglycans (Bespalov et al. 2011). Furthermore, the size of the human brain is much larger compared to rodent and monkey brains which exponentially decreases the area of tissue diffusion of GFLs. This issue can be partly solved by using GDNF and NRTN variants which have been shown to have better tissue diffusion and stability (Runeberg-Roos et al. 2016; Grondin et al. 2019) Therefore, these variants can be considered as potential candidates for treating PD patients. In addition, problems with tissue distribution can also be solved by using CDNF, a novel neurotrophic factor which has been shown to protect and restore dopamine neurons in various animal models of PD (Lindholm et al. 2007; Airavaara et al. 2012; Bäck et al. 2013; Subramanian 2013). Importantly, CDNF is currently in a phase I/II clinical trial in PD patients (NCT03295786). However, patients with an advanced stage of PD are predominantly selected for the clinical trials with NTFs due to the high invasiveness of the treatment. These patients have little, if any, dopamine neurons which can be rescued by GFLs. In addition, the impairment in axonal transport in late-stage patients might hinder retrograde transport of trophic factors necessary for the generation of cell survival signals. Therefore, early-stage PD patients should be selected to benefit from trophic factor therapy. The lack of GFL efficacy in clinical trials can also be due to low dose and/or low biological activity of a particular batch of the protein, which might have been insufficient to provide clinical improvement (Kirkeby and Barker 2019). Therefore, the design of future clinical trials with GFL-based drugs in PD patients has to be improved. In particular, special attention should be given to the selection of patients into treatment groups. In this regard, ethical pressure to choose late-stage PD patients and pharmacokinetic issues can be overcome by the use of systemically delivered small molecules targeting GFL receptors. These compounds, with improved tissue distribution, can also have the benefit of targeting both remaining axons of dopamine neurons in the putamen and their cell bodies in the SNpc.

## Neuropathic pain

Neuropathic pain (NP) is a long-lasting condition occurring as a result of a disease or lesion in the somatosensory system. It affects up to 10% of the population (Yawn et al. 2009; van Hecke et al. 2014), being more common in elderly people and often appears as a result of trauma, disease, or treatment. The prevalence of NP is expected to grow in the future because of population aging and increase in the number of people affected by conditions causing NP.

The current management of NP is unsatisfactory. Existing drugs provide adequate pain relief only in a subset of patients and their use is often accompanied by the development of tolerance and dependence. Moreover, neither of the existing treatments is able to eliminate the cause of the disease or in other words protect and restore the function of injured sensory neurons (Fig. 2). This indicates the urgent need for the development of novel treatments improving the condition of injured sensory neurons and their function in NP patients.

## GFLs in Neuropathic pain

Sensory neurons in dorsal root ganglia (DRGs) express receptors for several neurotrophic factors, in particular neurotrophin receptors TrkA, TrkB, and TrkC, and GFL receptors GFRα/RET (Orozco et al. 2001; Usoskin et al. 2015), and, therefore, respond to neurotrophins and GFLs. In healthy adult rodent DRGs, TrkA is expressed in approximately 40% of sensory neurons (McMahon et al. 1994; Orozco et al. 2001), TrkB and TrkC in 10–20% (McMahon et al. 1994; Orozco et al. 2001; Lin et al. 2011), RET in 60%, GFRα1 in 40%, GFRα2 in 30%, and GFRα3 in 20–40% of sensory neurons. Nerve injury increases the expression of RET (70%), GFRα1, and GFRα3, and downregulates GFRα2 expression (Bennett et al. 2000b; Wang et al. 2011). In healthy human DRGs, the percentage of cells expressing neurotrophin receptors and GFRα3 are very similar to rodents’, but the GFRα1–2/RET expression pattern is different: RET is expressed in approximately 80% of neurons, GFRα1 in 20%, and GFRα2 in 51% (Josephson et al. 2001). The distribution patterns of GFL receptors in the somatosensory system, and positive biological effects of GFLs, in particular GDNF and ARTN, in cultured sensory neurons, make them attractive targets for the development of analgesic treatments.

Neurotrophic factors play an important role in the development and maintenance of hypersensitivity and pain. While it is generally accepted that neurotrophins, in particular BDNF and NGF, are pronociceptive, reports on the biological effects of GFLs in the somatosensory system are rather contradictory. Both GDNF and ARTN were tested in animal models of neuropathic pain.

In injury-based models of experimental neuropathies, GDNF and ARTN prevented and reversed tactile and thermal hypersensitivity, and normalized the expression of various sensory neuron markers. GDNF was also shown to normalize electrophysiological properties of injured sensory neurons and the expression of sodium channels (Bennett et al. 1998; Boucher et al. 2000; Boucher and McMahon 2001; Wang et al. 2003, 2014, 2017; Hao et al. 2003; Gardell et al. 2003; Zwick et al. 2003; Pezet et al. 2006; Sakai et al. 2008; Hubbard et al. 2008; Takasu et al. 2011; Fukuoka and Noguchi 2015). In addition, intraperitoneally injected ARTN relieved herpes virus-induced mechanical hypersensitivity (Asano et al. 2006). Importantly, in the dorsal root crush model, systemically delivered ARTN promoted the regrowth of axons and also guided them into topographically correct regions of the spinal cord (Wang et al. 2008; Harvey et al. 2010). In these studies, ARTN also induced the regrowth of all types of sensory afferents into the spinal cord. The effects of neurotrophins in this model were quite different: they promoted the robust regrowth of only peptidergic fibers into the spinal cord in the absence of topographic targeting. Noteworthy, ARTN overexpressed from the lentiviral vector in DRGs failed to promote regrowth of axons into the spinal cord and the overexpression of this protein in the spinal cord failed to promote the regeneration of non-peptidergic sensory axons, while correctly targeting peptidergic ones (Kelamangalath et al. 2015). The mechanism of topographically correct targeting of regenerating axons by systemic ARTN is somewhat mysterious. The ARTN ligand-binding subunit, GFRα3 co-receptor, is expressed only in CGRP-positive, peptidergic sensory neurons in healthy organisms. Nerve lesions also stimulate the expression of GFRα3 in some non-peptidergic neurons. For example, it was shown that GFRα2-positive neurons switch phenotype after the nerve cut, shutting down GFRα2 expression and becoming GFRα3-positive (Wang et al. 2011). ARTN can also act via indirect mechanisms by, for example, increasing the expression of other axon guidance proteins, such as GDNF. It is also unclear why spinal overexpression and systemic delivery had different effects in non-peptidergic sensory neurons.

The effects of GFLs in inflammatory and cancer pain states in mice seem to be opposite to the data collected in nerve injury-based models. Inflammation and cancer modulated the expression of GFLs in both experimental animals (Toma et al. 2002; Hashimoto et al. 2005; Malin et al. 2006; Ikeda-Miyagawa et al. 2015; Ding et al. 2017) and humans (von Boyen et al. 2006; Ceyhan et al. 2007). The level of ARTN expression correlated with the severity of pain in chronic pancreatitis in humans (Ceyhan et al. 2007). Sequestration of GFLs with antibodies alleviated complete Freund’s adjuvant-induced mechanical hypersensitivity (Fang et al. 2003; Amaya et al. 2004; Thornton et al. 2013; Nencini et al. 2018) and cold allodynia (Lippoldt et al. 2016) in experimental animals and reduced electrical activity of bone marrow nociceptors in a model of carrageenan-induced bone pain (Nencini et al. 2019). However, lentiviral vector-mediated GDNF overexpression alleviated tumor-induced mechanical and thermal hyperalgesia (Ding et al. 2017).

Reports of GFL actions in healthy animals are inconsistent. In many studies conducted in rats, GFLs seem to have no influence on pain-like behavior (Boucher et al. 2000; Gardell et al. 2003; Ramer et al. 2003; Yoshida et al. 2011) although some authors report heat and mechanical hyperalgesia after repeated ARTN injections (Ikeda-Miyagawa et al. 2015) or single injection of a high dose of GDNF (Joseph and Levine 2010). At the same time in mice, GFL administration causes mainly thermal (Malin et al. 2006; Lippoldt et al. 2013), but in some studies also mechanical, hypersensitivity (Wang et al. 2018). Delivery route and administration schedule may play a role in hypersensitivity responses in GFL-treated healthy animals. While subcutaneous injections were well-tolerated, direct injections of GFLs into, for example, bone marrow, plantar surface, or intrathecal overexpression were pronociceptive. Also, single injection of ARTN in rats (Ikeda-Miyagawa et al. 2015) or subcutaneous administration on every other day (Gardell et al. 2003) produced no pain-like responses contrary to repeated every day administration (Ikeda-Miyagawa et al. 2015). Poor tissue distribution of GFLs could have resulted in a very high point concentration of the protein in the case of local delivery, which produced detrimental effects on the sensitivity to various pain stimuli. Importantly, pronociceptive effects of GFLs seem to be mainly short-term (although the data of Joseph and Levine 2010 contradict this statement), which may explain some discrepancies in experimental results.

The data collected from GFLs and GFL receptor knockin and knockout mice stress the importance of GFL signalling in the somatosensory system. Overexpression of GDNF in the skin could have altered mechanical sensitivity (contradictory results are presented in Zwick et al. 2003; Albers et al. 2006) in näive animals, but had no influence on thermal sensitivity, despite increased density of intraepidermal nerve fibers (Zwick et al. 2003). ARTN overexpression, accompanied by the increased number of nociceptors in DRGs, and expression of transient receptor potential (TRP)V1 and TRPA1 resulted in hypersensitivity to heat and noxious cold (Elitt et al. 2006; Shu-Ying et al. 2008). At the same time, knocking out RET in nociceptors resulted in hypersensitivity to cold (Golden et al. 2010). GFRα2 knockout mice are hypersensitive to thermal stimulation but have a reduced response to inflammatory pain in the formalin test and normal tactile sensitivity (Lindfors et al. 2006). GFRα3 knockout mice have a normal response to tactile and thermal stimuli, but fail to produce cold allodynia in response to inflammatory, traumatic, or chemotherapeutic nerve injury (Lippoldt et al. 2016). As GFLs are survival factors for nociceptors and play an important role in the development of the somatosensory system, it is not surprising that modulation of their expression or the expression of their receptors can alter thermal and mechanical sensitivity as a result of, for example, increased number of nociceptors in DRGs or reduced density of intraepidermal nerve fibers in the skin.

Pronociceptive effects of GFLs seem to be related to potentiation of ion channel protein TRPV1 and TRPA1 signalling (Malin et al. 2006) and overexpression of nAChR (Albers et al. 2014) reported in mice. At the same time in rats, one group reported downregulation of TRPA1 channel activity by GDNF (Yoshida et al. 2011).

In translational research, animal models are important for the preliminary stage before clinical trials, however it is even more important to understand which effects GFLs have in humans. ARTN was found to be safe and relatively well-tolerated in phase I/II clinical trials in patients with neuropathic pain (Rolan et al. 2015; Okkerse et al. 2016). In a randomized placebo-controlled double-blinded phase II clinical trial conducted in patients resistant to treatments with at least two standard analgesics, ARTN produced significant pain relief and improved sleep quality assessed by daily sleep interference (Backonja et al. 2017). Interestingly, a U-shaped dose-response curve was seen in patients treated with ARTN: the lowest dose produced the highest pain relief and the second most efficient dose was the highest one tested. Pharmacokinetics studies revealed a direct correlation between injected dose and ARTN concentration in serum; therefore, pharmacokinetic differences are unlikely to explain such dose-response relations. Since the concentration of ARTN in target tissue was not analyzed in this trial, the authors could not completely exclude that the variation in the drug accumulation in the nervous system of patients treated with different doses produced a U-shaped dose-response, although it does not seem to be very likely. Importantly, non-linear dose-response relations are not uncommon for neurotrophic factors. The previously published literature, including our findings, have shown that the survival-promoting effects of GFLs are dose-dependent, showing an inverted U-shaped dose-response curve (bell-shaped) (Hou et al. 1996; Mills et al. 2007; Planken et al. 2010; Saarenpää et al. 2017). Apart from pharmacokinetics, this can be explained by (i) negative feedback mechanisms in signalling cascades overactivated by high doses of GFLs; (ii) downregulation of GFL receptor expression; (iii) reduction in the number of cell surface receptors, as GFL binding leads to internalization of ligand-receptor complex with subsequent degradation or recycling; (iv) biochemical overload of the signalling system with ligand which causes the binding of each individual GFRα monomer to a molecule of a ligand leading to inability to dimerize and form a tetrameric receptor complex (Schlee et al. 2006); and (v) hyperactivation of phosphatases dephosphorylating RET and, thus, shutting down RET-dependent signalling (Yadav et al. 2020).

The main adverse effect of ARTN treatment in patients was pruritus, also headaches, changes in temperature perception, and rashes were reported. Most of the adverse effects were short-lasting and of mild-to-moderate severity. It is important to mention here that GFLs can signal via other receptors than RET, in particular NCAM and syndecan-3 (Fig. 1). Sensory neurons positive for ARTN’s cognate receptor GFRα3 express RET only in 30% of cases (Bennett et al. 2000a) and it was also shown that RET-negative TRPM8-positive neurons mediate cold sensitivity in experimental animals (Lippoldt et al. 2013, 2016). Moreover, GFRα3 co-receptor is expressed in Schwann cells, which do not express RET (Thai et al. 2019). This can explain some effects of GFLs in promotion of bone pain and altered temperature perception. Receptor expression patterns published by Usoskin and co-authors also support this conclusion and indicate that while the pruritus is mediated by RET, cold hypersensitivity can possibly be mediated by other receptors (Usoskin et al. 2015). ARTN also seems to have a role in migraine (Shang et al. 2016). However, it is unclear if systemically delivered ARTN could have triggered headache, as it fails to cross the blood-brain barrier.

All these data taken together indicate that GFLs and their receptors are important targets for neuronal repair in NP states. However, special attention should be given to the inflammatory status of the patients, delivery route and schedule, tissue localization, and expression pattern of GFL receptors. Therefore, compounds selectively targeting components of the GFL signalling complex can offer advantages in translational research, producing less side effects in NP patients compared to GFLs themselves.

## Targeting GFL receptors with small molecules for the development of therapeutics against Parkinson’s disease and neuropathic pain

GFL proteins play an important role in the survival and reparation of dopamine and sensory neurons and, therefore, hold promise for disease modification in PD and NP. However, GFLs themselves have many disadvantages as therapeutically used drugs. GFLs do not cross tissue barriers and have to be delivered directly to the action site, which means in, for example, PD patient delivery by complicated brain surgery. They poorly spread in tissues (Salvatore et al. 2006) which is caused by their high affinity to the extracellular matrix and cell surface proteoglycans, and therefore, they may fail to reach target neurons even if delivered to the correct place. Their production is complicated and expensive. The biological activity of the resulting protein can depend on the production system (Saarenpää et al. 2017) and vary between batches. In addition, it can be diminished if the protein is stored or handled under suboptimal temperatures (> 4 °C).

GFLs also target several receptors expressed rather ubiquitously in different cell types. This can result in undesirable side effects, such as cold allodynia in response to ARTN injections mediated by non-RET-related signalling or bone pain produced by the same protein in which Schwann cells expressing GFRα3 can contribute.

High point concentrations of GFLs produced as a result of delivery of extremely high concentrations into a specific tissue and relatively long half-life of GFL proteins or constant overexpression from viral vectors can be detrimental for the cells which is indirectly indicated by biphasic dose-response curves seen in, for example, clinical trials and in cultured cells.

The biphasic dose-response depends upon the endpoint measured. U-shaped dose-response curves display a strong effect of the drug at a low dose, less effect of a drug at an intermediate dose, and the second increase in effect at a high dose. Similarly, in the case of an inverted U-shaped dose-response curve, the effect of the drug at first increases with increase in dose and then decreases at higher concentrations (Calabrese and Baldwin 2001). Biphasic dose-response curves complicate clinical translation as well as interpretation of efficacy and side effect data in clinical trials.

Existing data for GFLs suggest that they show their effects in a narrow range of concentrations, being more effective in, for example, survival promoting at lower concentrations (Hou et al. 1996; Mills et al. 2007; Planken et al. 2010; Saarenpää et al. 2017). Individual variability in the expression level of GFL-binding proteins (e.g., components of the extracellular matrix) can further complicate interpretation of the data by producing differences in their concentrations in target tissues at the organism level. These issues, together with multiple sites of action and several receptors transmitting GFL signals, make the selection of safe and efficient doses of these proteins in clinical trials difficult.

Targeting GFL receptors with small molecules can help to overcome at least some limitations associated with clinical development of GFL proteins. Development, optimization, production, and storage of chemically based drugs are well-established by the pharmaceutical industry. Such compounds may spread in tissues well and cross tissue barriers allowing implementation of non-invasive delivery schemes.

The first molecule which was shown to bind GDNF’s co-receptor GFRα1 and promote the neurite outgrowth from neuroblastoma cells, XIB4035, was discovered by a Japanese group (Tokugawa et al. 2003). A follow-up study revealed that XIB4035 itself is unable to activate GFL receptors, but can enhance the effects of endogenous proteins (Hedstrom et al. 2014). Topical application of XIB4035 in animals with streptozotocin- or genetically induced small fiber neuropathy alleviated loss of thermal nociception, prevented the loss of intradermal nerve fibers and Remark bundles, and also restored the density of IB4-positive axons in the spinal cord. In this study, XIB4035 produced no side effects, did not alter heat sensitivity in näive mice, and had no influence on mechanical sensitivity in any treatment groups (Hedstrom et al. 2014). At the same time, the other group reported the development of mechanical allodynia in näive mice in response to XIB4035 delivered intravertebrally while thermal sensitivity remained unaffected (Hsieh et al. 2018). XIB4035 has not been tested in PD models.

High-throughput screening of the libraries of chemical compounds led to the discovery of two classes of structurally unrelated selective RET agonists (Bespalov et al. 2016; Sidorova et al. 2017; Mahato et al. 2019a, b; Jmaeff et al. 2020). One class of these compounds, BT compounds, was shown to support the survival of naïve and toxin-challenged cultured dopamine neurons (Mahato et al. 2019a) and promote neurite outgrowth from cultured sensory neurons (Sidorova et al. 2017). These compounds also alleviated behavioral manifestations of PD (Mahato et al. 2019b; Renko et al., manuscript) and neuropathy-induced pain-like behavior in animal models (Bespalov et al. 2016; Sidorova et al. 2017; Viisanen et al., manuscript). BT compounds also stimulated release of dopamine in the brain of experimental animals and protected and restored sensory neuron phenotypes in animal models of NP. Importantly, these compounds were able to cross the blood-brain barrier and could, therefore, be delivered systemically in patients with PD providing potential treatment options for the most responsive patient population (early-stage PD patients), which are now mainly excluded from clinical trials due to ethical restrictions. BT compounds delivered systemically did not influence thermal and mechanical sensitivity and seem to be well-tolerated by experimental animals (Sidorova et al. 2017), which is in line with some reports for GFL (Boucher et al. 2000; Gardell et al. 2003). The absence of hyperalgesic and allodynic effects of BT compounds can also be explained by their short half-life and quick elimination, better tissue diffusion resulting in lower point concentrations, and lower efficacy compared to GFLs. Further optimization of BT compounds for their improved biological activity and drug-like properties can eventually convert them into the first-in-class disease-modifying therapeutics against PD and NP.

The second discovered class of selective RET agonists is yet to be tested in dopamine and sensory systems. These compounds support retinal cells in tissue explants and have better pharmacological characteristics compared to the BT family (Saragovi et al. 2014; Jmaeff et al. 2020). They certainly deserve testing in PD and NP models in the future.

A molecular modelling approach led to the discovery of a class of small molecules selectively targeting GFRα co-receptors and weakly activating RET (Ivanova et al. 2018). Their biological activity is insufficient to test them in primary neurons or animal models of PD and NP. However, their further development can lead to another approach in supporting the survival and function of GFL-dependent neurons in PD and NP.

## Conclusions and future perspective

PD and NP are characterized by malfunction and loss of dopamine and sensory neurons, respectively. The lack of drugs able to protect and restore these neuronal populations produces a significant challenge for the management of these diseases. Increasing prevalence of these conditions in the aging world population makes the development of novel treatments against PD and NP a burning need in current society. GFLs support dopamine and sensory neurons in the organism and are attractive but translationally complicated therapeutic options for the development of disease-modifying treatments against PD and NP. Multiple receptors, pleiotropic effects, biphasic dose-response, delivery, bioavailability, and dosage issues impede the clinical use of GFL proteins. Small drug-like molecules selectively targeting specific receptors or co-receptors in the GFL receptor complex may be a better alternative for translational research.

A few small molecular weight GFL receptor complex agonists have been discovered so far. The data from in vitro studies and animal models of PD and NP indicate that these compounds have promising properties for further preclinical development. At the moment, it is unclear which one of the GFL receptor-targeting strategies is the best for the development of the drugs against PD and NP. GFL signalling modulators such as XIB4035 can offer more physiological activation of RET-dependent cellular processes, but require the presence of an endogenous ligand to produce the effect. It can be problematic in patients with PD and nerve injury-induced NP states, because in these cases, the connection between neurons and GFL-producing tissues is impaired or lost. Direct RET agonists can target cells independently of the GFRα co-receptor expression pattern, thus affecting a number of neuronal subtypes. They also should not influence certain cells responsive to GFLs which do not express RET, e.g., Schwann cells (Thai et al. 2019) or TRPM8-positive neurons, whose activation is solely responsible for the development of cold allodynia upon ARTN treatment (Lippoldt et al. 2013, 2016). At the same time, RET is expressed in several non-neuronal tissues, such as testicles or developing kidneys; therefore, direct RET agonists can have effects in these cell types. Similarly, compounds targeting GFRα co-receptors can produce unwanted effects in cells lacking RET but expressing a co-receptor. Further studies are needed to clarify these issues and develop clinically safe small molecules targeting GFL receptors.

## Abbreviations

ARTN: artemin
CDNF: cerebral dopamine neurotrophic factor
GFLs: GDNF family ligands
GDNF: glial cell line-derived neurotrophic factors
GFRΑL: GDNF family receptor alpha-like
GFRα: GDNF family receptor alpha
GDF15: growth differentiation factor-15
MANF: mesencephalic astrocyte-derived neurotrophic factor
NRTN: neurturin
NP: neuropathic pain
PSPN: persephin
PD: Parkinson’s disease
RET: rearranged during transfection
SNpc: substantia nigra pars compacta
